# Diabetes Mellitus and the Benefit of Antiresorptive Therapy on Fracture Risk

**DOI:** 10.1002/jbmr.4697

**Published:** 2022-10-07

**Authors:** Richard Eastell, Eric Vittinghoff, Li‐Yung Lui, Susan K. Ewing, Ann V. Schwartz, Douglas C. Bauer, Dennis M. Black, Mary L. Bouxsein

**Affiliations:** ^1^ Department of Oncology and Metabolism University of Sheffield Sheffield UK; ^2^ Department of Epidemiology & Biostatistics University of California, San Francisco San Francisco CA USA; ^3^ Research Institute California Pacific Medical Center San Francisco CA USA; ^4^ Department of Medicine University of California, San Francisco San Francisco CA USA; ^5^ Center for Advanced Orthopedic Studies, Beth Israel Deaconess Medical Center and Department of Orthopedic Surgery Harvard Medical School Boston MA USA

**Keywords:** BIOCHEMICAL MARKERS OF BONE REMODELING, DXA, CLINICAL TRIALS, OSTEOPOROSIS, ANTIRESORPTIVES

## Abstract

Type 2 diabetes (T2D) is associated with increased risk of fractures. However, it is unclear whether current osteoporosis treatments reduce fractures in individuals with diabetes. The aim of the study was to determine whether presence of T2D influences the efficacy of antiresorptive treatment for osteoporosis using the Foundation for the National Institutes of Health (FNIH)–American Society for Bone and Mineral Research (ASBMR)–Study to Advance Bone Mineral Density (BMD) as a Regulatory Endpoint (SABRE) cohort, which includes individual patient data from randomized trials of osteoporosis therapies. In this study we included 96,385 subjects, 6.8% of whom had T2D, from nine bisphosphonate trials, two selective estrogen receptor modulator (SERM) trials, two trials of menopausal hormone therapy, one denosumab trial, and one odanacatib trial. We used Cox regression to obtain the treatment hazard ratio (HR) for incident nonvertebral, hip, and all fractures and logistic regression to obtain the treatment odds ratio (OR) for incident morphometric vertebral fractures, separately for T2D and non‐DM. We used linear regression to estimate the effect of treatment on 2‐year change in BMD (*n* = 49,099) and 3‐month to 12‐month change in bone turnover markers (*n* = 12,701) by diabetes status. In all analyses, we assessed the interaction between treatment and diabetes status. In pooled analyses of all 15 trials, we found that diabetes did not impact treatment efficacy, with similar reductions in vertebral, nonvertebral, all, and hip fractures, increases in total hip and femoral neck BMD, and reductions in serum C‐terminal cross‐linking telopeptide (CTX), urinary *N*‐telopeptide of type I collagen/creatinine (NTX/Cr) and procollagen type 1 N propeptide (P1NP) (all interactions *p* > 0.05). We found similar results for the pooled analysis of bisphosphonate trials. However, when we considered trials individually, we found a few interactions within individual studies between diabetes status and the effects of denosumab and odanacatib on fracture risk, change in BMD or bone turnover markers (BTMs). In sum, these results provide strong evidence that bisphosphonates and most licensed antiresorptive drugs are effective at reducing fracture risk and increasing BMD irrespective of diabetes status. © 2022 The Authors. *Journal of Bone and Mineral Research* published by Wiley Periodicals LLC on behalf of American Society for Bone and Mineral Research (ASBMR).

## Introduction

Increased fracture risk is a recognized complication of type 2 diabetes mellitus (T2D) with a 33% increase in hip fracture and a 19% increase in nonvertebral fracture risk compared to those without diabetes, based on a recent meta‐analysis.^(^ ^1^ ^)^ The increased risk of fracture in T2D is associated with normal or slightly increased bone mineral density (BMD)^(^ ^2^ ^)^ and normal or low bone turnover markers (BTMs).^(^ ^3^ ^)^

Low bone turnover in patients with T2D could result in reduced efficacy of anti‐resorptive drugs. However, few studies have examined the efficacy of anti‐osteoporosis medications in T2D. Indeed, there have been no clinical trials of anti‐resorptive drugs in T2D specifically. Many large trials of anti‐resorptive drugs in postmenopausal osteoporosis have included patients with T2D. However, only a few post hoc analyses have utilized the data from these trials to assess the efficacy of anti‐resorptive treatments in those with T2D. In the Fracture Intervention Trial (FIT), alendronate had similar effects on lumbar spine and total hip BMD over 3 years in women without and with T2D,^(^ ^4^ ^)^ but fracture efficacy was not assessed. In the Multiple Outcomes of Raloxifene Evaluation (MORE) study, raloxifene appeared to provide greater reductions in vertebral fracture in women with than without T2D, but there were only 45 patients with T2D.^(^ ^5^ ^)^ In the Raloxifene Use for The Heart (RUTH) study, raloxifene had similar effects on vertebral fracture reduction in women with and without T2D.^(^ ^6^ ^)^ In the Fracture Reduction Evaluation of Denosumab in Osteoporosis Every 6 Months (FREEDOM) study, denosumab had similar efficacy for vertebral fracture reduction in those with and without T2D but appeared to increase the risk of nonvertebral fractures in women with T2D while decreasing the risk in those without T2D.^(^ ^7^ ^)^

Given these inconsistent and incomplete findings, it is important to examine systematically whether effects of anti‐resorptive treatments on fracture, BMD, and BTM differ in patients with T2D. In the Foundation for the National Institutes of Health (FNIH)–American Society for Bone and Mineral Research (ASBMR)–Study to Advance Bone Mineral Density (BMD) as a Regulatory Endpoint (SABRE) project we obtained a unique dataset of individual patient data from randomized, placebo‐controlled trials of osteoporosis therapies.^(^ ^8^ ^)^ Using this dataset, we previously conducted a meta‐regression of the association between treatment‐related changes in BMD and fracture risk reduction. In the current analyses, we use this dataset of individual patient data to test whether the effects of antiresorptive treatments on fracture risk, change in BMD and change in BTM are similar between those with and without diabetes.

## Subjects and Methods

As described,^(^ ^8^ ^)^ we did a systematic search of published literature to identify randomized, controlled trials (RCTs) of osteoporosis drugs with fracture as the outcome. We checked databases including PubMed, Embase, and Cochrane for publications between 1985 and 2018 in English using the search terms “fracture, BMD, bone mineral density, and required RCT or synonyms”. We excluded small studies and studies of patients with certain conditions (eg, glucocorticoid‐induced osteoporosis).^(^ ^8^ ^)^

We attempted to collect from trial sponsors the following: complete data files, individual patient data, and study documentation. We included trials of approved osteoporosis medications, as well as trials of drugs for which approval was not sought or received. We used a standardized template including uniform fracture definitions and standardized BMD conversions. Some studies were not included in the FNIH‐SABRE databases as the sponsors were unable or unwilling to provide the data. Of the 23 trials included in the FNIH‐SABRE databases, 15 were included in these analyses (Table 1). Trials of anabolic therapies (*n* = 3) were excluded. An additional five trials were excluded due to no diabetes mellitus (DM) data or only family DM information. We allowed different definitions of T2D across trials. The definition of diabetes was provided by the trial sponsor. Subjects without information on diabetes status (*n* = 4986) were excluded from these analyses.

**Table 1:** DM Status by Trial in SABRE Study

Trial	Drug class	Study drug	*N* in non‐DM	*N* in T2D
FIT I Black 1996^(^ ^9^ ^)^	Bisphosphonate	Alendronate	1955	72
FIT II^(^ ^10^ ^)^	Bisphosphonate	Alendronate	4286	146
BONE^(^ ^11^ ^)^	Bisphosphonate	Ibandronate	2831	98
IBAN IV^(^ ^12^ ^)^	Bisphosphonate	Ibandronate (intravenous)	2835	25
HIP^(^ ^13^ ^)^	Bisphosphonate	Risedronate	8816	515
VERT‐MN^(^ ^14^ ^)^	Bisphosphonate	Risedronate	791	23
VERT‐NA^(^ ^15^ ^)^	Bisphosphonate	Risedronate	1568	60
HORIZON PFT^(^ ^16^ ^)^	Bisphosphonate	Zoledronic acid (intravenous)	7234	502
HORIZON RFT Lyles 2007^(^ ^17^ ^)^	Bisphosphonate	Zoledronic acid (intravenous)	2092	35
LOFT^(^ ^18^ ^)^	Odanacatib	Odanacatib	14,302	1769
WHI‐E^(^ ^19^ ^)^	Hormone therapy	Hormone therapy	9682	1054
WHI‐EP^(^ ^20^ ^)^	Hormone therapy	Hormone therapy	15,626	971
FREEDOM^(^ ^21^ ^)^	Denosumab	Denosumab (subcutaneous)	7192	596
PEARL^(^ ^22^ ^)^	SERMs	Lasofoxifene	8051	505
MORE^(^ ^23^ ^)^	SERMs	Raloxifene	2585	168

BONE = Oral Ibandronate Osteoporosis Vertebral Fracture Trial in North America and Europe; DM = diabetes mellitus; FIT = Fracture Intervention Trial; FREEDOM = Fracture Reduction Evaluation of Denosumab in Osteoporosis Every 6 Months; HIP = Hip Intervention Program Study Group; HORIZON PFT = Health Outcomes and Reduced Incidence with Zoledronic Acid Once Yearly Pivotal Fracture Trial; HORIZON RFT = Health Outcomes and Reduced Incidence with Zoledronic Acid Once Yearly Recurrent Fracture Trial; LOFT = Long‐term Odanacatib Fracture Trial; MORE = Multiple Outcomes of Raloxifene Evaluation; PEARL = Postmenopausal Evaluation and Risk‐Reduction with Lasofoxifene Study; SERM = selective estrogen receptor modulator; VERT‐MN = Vertebral Efficacy with Risedronate Therapy, Multinational Trial; VERT‐NA = Vertebral Efficacy with Risedronate Therapy, North American Trial; WHI‐E = Women’s Health Initiative, Estrogen Arm; WHI‐EP = Women’s Health Initiative, Estrogen‐Progestin Arm.

We created standardized fracture definitions across all trials for four categories of fractures: vertebral, nonvertebral, hip, and “all” fracture. For vertebral fractures, we used the individual study definitions based on comparing baseline with follow‐up lateral spinal radiographs. Vertebral fracture definition was based on quantitative morphometry,^(^ ^24^ ^)^ semiquantitative assessment,^(^ ^25^ ^)^ or a combination of these criteria. “All fractures” is the composite of nonvertebral and clinical or morphometric vertebral fractures. We excluded fractures due to major trauma, by which we meant trauma sufficient to cause a fracture in a young, healthy person. However, we included all fractures reported when trauma information was not available. We also excluded fractures of the fingers, toes and face. All subjects included in this report had information on nonvertebral, hip, and all fractures.

BMD was measured in 62,178 participants at baseline in all of the included trials. Because BMD was measured by different devices across the trials (Hologic, Bedford, MA; GE Lunar, Madison, WI; and Norland Corporation, Fort Atkinson, WI), we used standard equations to convert from Lunar and Norland to Hologic for the total hip, femoral neck, and lumbar spine^(^ ^26^, ^27^ ^)^ to generate standardized BMD (mg/cm^2^) values that were comparable across dual‐energy X‐ray absorptiometry (DXA) devices. We used the lumbar spine region L_1_–L_4_ when available, otherwise we used L_2_–L_4_. We used the National Health and Nutrition Examination Survey (NHANES) data^(^ ^28^ ^)^ to calculate the baseline femoral neck BMD *T*‐score for each trial, using the non‐Hispanic white female reference data.

BTMs were measured in 14,079 participants in 11 of the included trials at baseline. BTMs were measured in serum (procollagen type 1 N propeptide [P1NP] and C‐terminal cross‐linking telopeptide [CTX]) or second morning void urine (urinary *N*‐telopeptide of type I collagen/creatinine [NTX/Cr]). The assay for P1NP included a radioimmunoassay (Orion Diagnostica, Espoo, Finland) and an automated immunoassay analyzer method (Roche Elecsys 2010; Roche, Penzburg, Germany). The assays for sCTX included an enzyme‐linked immunosorbent assay (ELISA) (CrossLaps; Nordic Bioscience Diagnostics AS, Herlev, Denmark) and an automated immunoassay analyzer (Roche Elecsys 2010; Roche, Penzburg, Germany). The assays for urine NTX included an ELISA (Osteomark; Ostex International Inc, Seattle, WA, USA) and an automated immunoassay analyzer method (Vitros ECi; Ortho Clinical Inc, Rochester, NY, USA).

### Statistical Analysis

To estimate and compare the effects of treatment on fracture risk separately for T2D and non‐DM, we used Cox proportional hazard models for time to first fracture for all, nonvertebral, and hip fractures, and logistic models for vertebral fractures, for which the exact time to event was unknown. These stratified models included an indicator for treatment as well as indicators for trial, allowing different baseline risks in each. The T2D and non‐DM groups were then combined to test the two‐way interaction between treatment and T2D status; the interaction models included indicators for trial, treatment, and T2D status, and the interaction between treatment and T2D status, allowing us to compare estimated treatment effects in T2D and non‐DM. Models were first estimated using the individual patient data (IPD) pooled across all studies, then repeated using bisphosphonate trials only.

In additional analyses within trials, we estimated and compared the effects of treatment on fracture risk in T2D and non‐DM, using the methods for the overall analysis to assess the evidence for interaction between treatment and T2D within each trial. Results are presented in forest plots. To evaluate evidence for heterogeneity across studies, we used the overall pooled IPD to estimate Cox and logistic models that included indicators for study, treatment, and T2D versus non‐DM, and all two‐way and three‐way interactions between these factors. The test for three‐way interaction assesses heterogeneity in the T2D versus non‐DM comparisons across studies.

The same approach, in this case using linear models, was used to estimate and compare the effects of treatment on changes in BMD and BTM in T2D and non‐DM, overall and within each study. Results are presented as difference (active − placebo) in mean percentage change (95% confidence interval [CI]) in BMD at 24 months and in BTM at 3 to 12 months. Changes in BTM measures were skewed so we used the log ratio of BTM (log[follow‐up BTM/baseline BTM]) in models, with results back‐transformed for interpretability.

All analyses were by intent to treat, without regard to adherence to treatment, and were performed using SAS software (version 9.4, SAS Institute Inc., Cary, NC, USA).

## Results

The baseline characteristics of 96,385 subjects who had information about their diabetes status and fracture outcomes are shown in Table 2. Of these, 6.8% had T2D. Fifteen RCTs met the inclusion criteria (Table 1), including nine trials of bisphosphonates.

**Table 2:** Baseline Characteristics

Characteristic	Non‐DM (*N* = 89,846)	T2D (*N* = 6539)	*p*
Age (years), mean ± SD	69.3 ± 7.8	70.0 ± 7.5	<0.0001
Female (%)	99.4	99.9	<0.0001
BMI (kg/m^2^), mean ± SD	26.3 ± 5.0 (*n* = 89,467)	28.8 ± 6.0 (*n* = 6515)	<0.0001
Prevalent vertebral fracture (%)	46.9 (*n* = 59,915)	41.2 (*n* = 4328)	<0.0001
Previous nonvertebral fracture (%)	22.7 (*n* = 37,776)	21.9 (*n* = 2748)	0.31
Total hip *T*‐score, mean ± SD	−2.07 ± 0.84 (*n* = 53,941)	−2.03 ± 0.92 (*n* = 3951)	0.005
Femoral neck *T*‐score, mean ± SD	−2.37 ± 0.70 (*n* = 58,025)	−2.41 ± 0.77 (*n* = 4140)	0.003
Lumbar spine *T*‐score, mean ± SD	−2.65 ± 1.13 (*n* = 48,710)	−2.45 ± 1.25 (*n* = 3582)	<0.0001
CTX (ng/mL), mean ± SD	0.37 ± 0.20 (*n* = 9201)	0.34 ± 0.24 (*n* = 458)	<0.0001
P1NP (ng/mL), mean ± SD	53.7 ± 24.2 (*n* = 11,406)	47.9 ± 24.3 (*n* = 566)	<0.0001
NTX/Cr (nmol/mmol), mean ± SD	63.2 ± 43.1 (*n* = 5672)	51.4 ± 43.1 (*n* = 321)	<0.0001

BMI = body mass index; CTX = serum C‐terminal cross‐linking telopeptide; NTX/Cr = urinary *N*‐telopeptide of type I collagen/creatinine; P1NP = serum procollagen type I N‐propeptide.

At baseline, subjects with T2D (Table 2) were, on average, slightly older (mean 70 versus 69 years), with higher body mass index (BMI), higher total hip BMD (THBMD), lower femoral neck BMD (FNBMD), fewer vertebral fractures, and lower BTMs, whether by CTX, P1NP, or NTX/Cr, than those without DM.

Considering all trials together, the reduction in fracture risk, comparing treatment and placebo groups, was similar for those without or with T2D (all *p* for interaction >0.05) (Table 3). Limiting to only studies of bisphosphonates (oral or iv), the findings were similar (Table 4). The key estimates in these tables are the 95% CIs for the interaction hazard ratio (HR) or odds ratio (OR). The 95%CIs include one, and thus do not show that the treatment effects differ between participants with and without T2D. Also, we estimated the minimal detectable interaction effects for vertebral, nonvertebral, all, and hip fractures and these were 1.98, 1.48, 1.32, and 13.8, respectively. None of the interaction effect estimates or corresponding confidence bounds was close to these numbers.

**Table 3:** Pooled Analyses of Anti‐Fracture Treatment Efficacy in Non‐DM and T2D in 15 Trials of Anti‐Resorptive Medications

	Non‐DM		T2D			
Fracture type	Treatment effect HR or OR (95% CI)	% with fracture (*n*/*N*)	Treatment effect HR or OR (95% CI)	% with fracture (*n*/*N*)	Interaction HR or OR (95% CI)	Interaction *p* ^a^
Vertebral	0.52 (0.49–0.56)	7.3 (3947/54,193)	0.48 (0.36–0.63)	5.7 (215/3787)	0.91 (0.68–1.22)	0.53
Nonvertebral	0.82 (0.78–0.85)	9.6 (8647/89,846)	0.86 (0.74–1.00)	10.2 (666/6539)	1.05 (0.89–1.22)	0.58
All	0.72 (0.69–0.74)	13.9 (12,478/89,846)	0.74 (0.64–0.84)	13.5 (880/6539)	1.02 (0.89–1.17)	0.80
Hip	0.68 (0.61–0.77)	1.3 (1202/89,846)	0.82 (0.57–1.16)	1.9 (125/6539)	1.20 (0.83–1.74)	0.33

All results are adjusted for trial.

^a^ Two‐way interaction: Treatment * Diabetes status.

**Table 4:** Pooled Analyses of Anti‐Fracture Treatment Efficacy in non‐DM and T2D in Nine Bisphosphonate Trials

	Non‐DM		T2D			
Fracture type	Treatment effect HR or OR (95% CI)	% with fracture (*n*/*N*)	Treatment effect HR or OR (95% CI)	% with fracture (*n*/*N*)	Interaction HR or OR (95% CI)	Interaction *p* ^a^
Vertebral	0.56 (0.51–0.61)	8.4 (2089/24,910)	0.38 (0.23–0.64)	7.0 (74/1060)	0.73 (0.44–1.20)	0.21
Nonvertebral	0.88 (0.82–0.94)	9.6 (3094/32,408)	0.77 (0.56–1.06)	10.3 (152/1476)	0.90 (0.65–1.24)	0.51
All	0.74 (0.70–0.79)	15.4 (4982/32,408)	0.64 (0.49–0.83)	14.7 (217/1476)	0.87 (0.67–1.15)	0.32
Hip	0.69 (0.58–0.83)	1.6 (517/32,408)	1.13 (0.58–2.20)	2.5 (37/1476)	1.69 (0.85–3.35)	0.14

All results are adjusted for trial.

^a^ 2‐way interaction: Treatment * Diabetes status.

When considering all trials together, the differences in changes in BTM and BMD expressed as a percentage, comparing treatment and placebo groups, did not differ (all *p* for interaction >0.05) between those with and without T2D (Table 5). We found similar results when limiting the analyses to the bisphosphonate trials (Table 6).

**Table 5:** Comparison of Effect of Treatment on Changes in BMD and BTM in Non‐DM and T2D in 13 Trials

	Non‐DM		T2D		
Parameter	*N*	Mean (95% CI)	*N*	Mean (95% CI)	Interaction *p* ^a^
% Difference in BMD change (active − placebo) at 24 months					
Total hip	43,258	3.85 (3.77–3.93)	3001	3.86 (3.54–4.18)	0.95
Femoral neck	45,966	3.45 (3.35–3.54)	3124	3.38 (2.98–3.78)	0.67
Lumbar spine	33,274	4.61 (4.51–4.72)	2266	4.37 (3.94–4.79)	0.21
% Difference in BTM change at 3 to 12 months (active − placebo)					
CTX	8056	−52.3 (−53.6, −51.0)	367	−53.2 (−59.5, −45.9)	0.69
P1NP	9642	−46.2 (−47.2, −45.1)	447	−40.6 (−45.8, −34.9)	0.06
NTX/Cr	4946	−41.8 (−43.6, −39.9)	246	−40.8 (−48.1, −32.5)	0.86

All results are adjusted for trial.

BMD = bone mineral density; BTM = bone turnover marker; CTX = serum C‐terminal cross‐linking telopeptide; NTX/Cr = urinary *N*‐telopeptide of type I collagen/creatinine; P1NP = serum procollagen type I N‐propeptide.

^a^ Two‐way interaction: Treatment * Diabetes status.

**Table 6:** Comparison of Effect of Treatment on Changes in BMD and BTM in non‐DM and T2D in Nine Bisphosphonate Trials

	Non‐DM		T2D		
	*N*	Mean (95% CI)	*N*	Mean (95% CI)	Interaction *p* ^a^
% Difference in BMD change (active − placebo) at 24 months					
Total hip	17,612	3.66 (3.53–3.78)	716	4.08 (3.42–4.75)	0.20
Femoral neck	19,869	2.89 (2.74–3.04)	791	3.13 (2.27–3.99)	0.57
Lumbar spine	13,054	4.33 (4.17–4.49)	385	4.62 (3.76–5.47)	0.46
% Difference in BTM change at 3 to 12 months (active − placebo)					
CTX	5789	−52.1 (−53.7, −50.5)	197	−51.1 (−59.8, −40.5)	0.88
P1NP	7094	−50.3 (−51.4, −49.2)	265	−44.2 (−50.6, −36.9)	0.09
NTX/Cr	3774	−37.6 (−39.8, −35.2)	119	−41.5 (−51.5, −29.3)	0.53

BMD = bone mineral density; BTM = bone turnover marker; CTX = serum C‐terminal cross‐linking telopeptide; NTX/Cr = urinary *N*‐telopeptide of type I collagen/creatinine; P1NP = serum procollagen type I N‐propeptide.

All results are adjusted for trial.

^a^ 2‐way interaction: Treatment * Diabetes status.

**Fig. 1:**
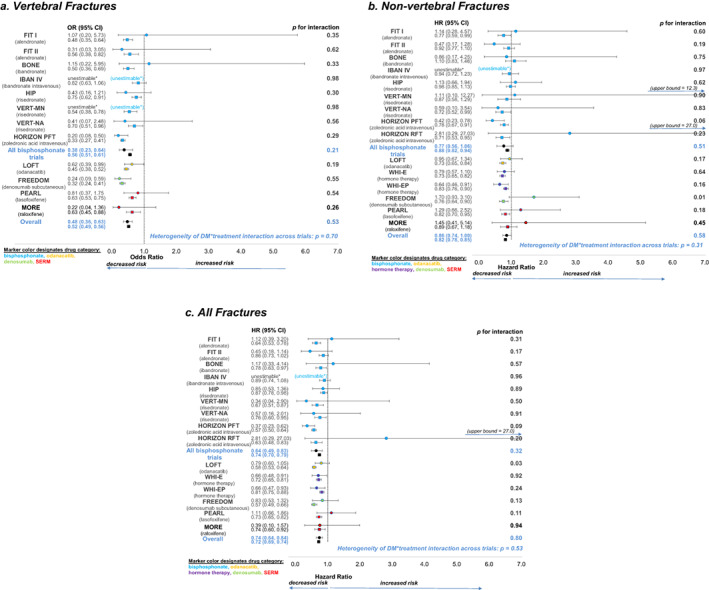
Forest plots showing the effects of treatment on fracture risk in T2D (solid circle) and non‐DM (solid square). a. vertebral fractures, b. non‐vertebral fractures and c. all fractures. The *p* values for T2D status*treatment interaction for each trial, the overall effects, and the *p* value for heterogeneity of T2D status*treatment interaction across trials are all shown. *unestimable: no fractures in active and/or placebo groups in T2D.

**Fig. 2:**
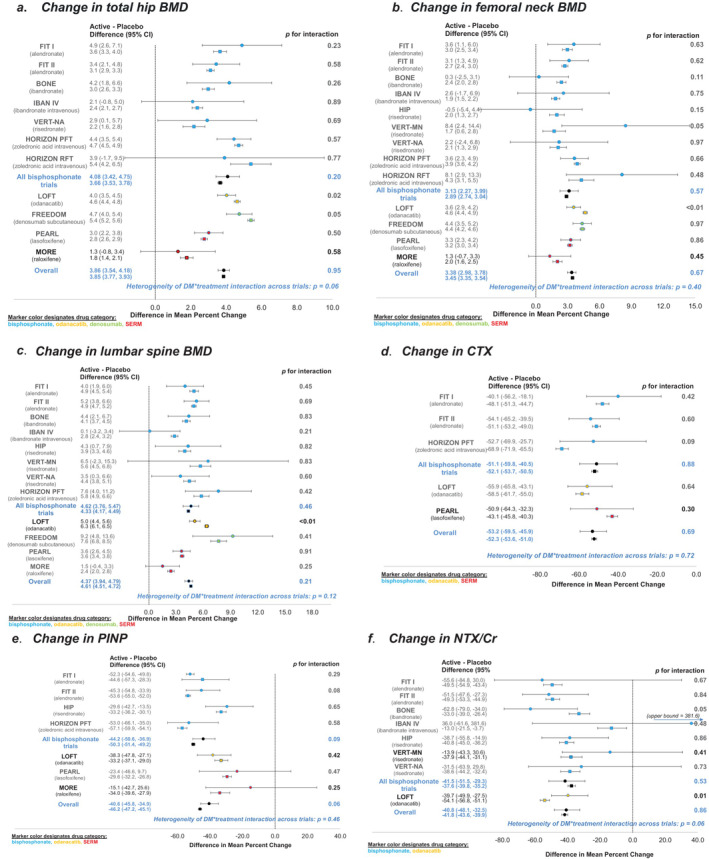
Forest plots showing the effects of treatment on change in BMD and BTM in T2D (solid circle) and non‐DM (solid square). a. change in total hip BMD, b. change in femoral neck BMD, c. change in lumbar spine BMD and d. change in CTX, e. change in P1NP, f. change in NTX/Cr. The *p* values for T2D status*treatment interaction for each trial, the overall effects, and the *p* value for heterogeneity of T2D status*treatment interaction across trials are all shown.

We present the results from each trial as forest plots. Figures 1a to c includes fracture outcomes, and Figures 2a to f. presents changes in BMD and BTM. For each trial, the plots provide an estimate of the effect of treatment in T2D and in non‐DM separately and a *p* value for interaction (treatment * T2D). The plots also include the estimate for the treatment effect in T2D and in non‐DM separately, across all trials and across all bisphosphonate trials, a *p* value for interaction for the overall effects, and a *p* value for heterogeneity of the treatment * T2D interactions across all trials. In most individual trials, there was no significant interaction of treatment with diabetes status for any of the outcomes. However, there were a few exceptions. For nonvertebral fracture, there was a significant interaction between T2D status and denosumab treatment (FREEDOM trial) (*p* = 0.01), with an increase in fracture risk in T2D on denosumab treatment, whereas fracture risk was reduced in non‐DM on treatment. For all fracture, there was a significant interaction between T2D status and odanacatib treatment (Long‐term Odanacatib Fracture Trial [LOFT]) (*p* = 0.03), with lower efficacy in T2D versus non‐DM, HR 0.79 (95% CI, 0.60 to 1.05) for all fracture for T2D on treatment versus HR 0.58 (95% CI, 0.53 to 0.64) for non‐DM on treatment.

We did not show the data for hip fractures as forest plots by individual trial because there were few hip fractures in most trials, particularly in the T2D group due to the limited number of subjects. However, there was one significant interaction term for an individual trial, and this was the Hip Intervention Program (HIP) trial of risedronate.^(^ ^13^ ^)^ The interaction term had a *p* value of 0.02, and the HR for hip fractures in patients with T2D was 3.1 (95% CI, 0.9 to 10.6), whereas the HR for hip fracture in patients without T2D was 0.68 (95% CI, 0.51 to 0.91). However, there were only three subjects with T2D in the placebo group so this might be a false positive.

For change in total hip BMD, there was a significant interaction with a lower increase in BMD in T2D compared to non‐DM for denosumab (*p* = 0.05) and odanacatib (*p* = 0.02). For femoral neck and lumbar spine BMD, there was a significant interaction with a lower increase in BMD in T2D for odanacatib treatment compared to the effect of treatment in non‐DM (*p* < 0.01).

For change in BTM, there were no significant interactions for individual trials for CTX or P1NP. However, for odanacatib treatment, there was less reduction in NTX for T2D compared to non‐DM (*p* = 0.01).

Finally, we have included the observed rates of each of the types of fractures, defined as the number of fractures per 1000 person‐years, along with the incident rate ratio to provide complete data for comparison of T2D to non‐DM (Tables 7, 8, 9, 10).

**Table 7:** Observed Percentage with Incident Vertebral Fracture by Trial

		Non‐DM			T2D		
Trial	Trial length (months)	Active (%)	Placebo (%)	RR	Active (%)	Placebo (%)	RR
FIT I	36	7.9	15.2	0.52	9.4	8.8	1.06
FIT II	48	2.1	3.7	0.57	1.4	4.5	0.32
BONE	36	4.3	8.2	0.52	9.5	8.3	1.14
IBAN IV	36	9.0	10.8	0.84	5.3	0	—
HIP	36	9.1	11.8	0.78	4.7	10.2	0.46
VERT‐MN	36	19.1	30.4	0.63	0	25	—
VERT‐NA	36	11.2	15.3	0.73	8.7	19.0	0.46
HORIZON PFT	36	4.0	11.3	0.36	2.5	11.5	0.22
LOFT	60	4.2	8.9	0.47	3.9	6.2	0.64
FREEDOM	36	2.3	7.1	0.33	2.1	8.5	0.25
PEARL	60	6.3	9.7	0.65	5.6	6.9	0.82
MORE	36	5.5	8.4	0.65	1.8	7.7	0.23

Exact time to fracture cannot be assessed for incident morphometric vertebral fractures. Incident vertebral fracture status was assessed at multiple time points throughout most trials but only at the final study visit for other trials. For all trials, the overall incident vertebral fracture status was set to the fracture status as of the final study visit (ie, it was set to yes if an incident fracture occurred at any point during the trial).

BONE = Oral Ibandronate Osteoporosis Vertebral Fracture Trial in North America and Europe; FIT = Fracture Intervention Trial; FREEDOM = Fracture Reduction Evaluation of Denosumab in Osteoporosis Every 6 Months; HIP = Hip Intervention Program Study Group; HORIZON PFT = Health Outcomes and Reduced Incidence with Zoledronic Acid Once Yearly Pivotal Fracture Trial; LOFT = Long‐term Odanacatib Fracture Trial; MORE = Multiple Outcomes of Raloxifene Evaluation; PEARL = Postmenopausal Evaluation and Risk‐Reduction with Lasofoxifene Study; RR = rate ratio or relative risk; VERT‐MN = Vertebral Efficacy with Risedronate Therapy, Multinational Trial; VERT‐NA = Vertebral Efficacy with Risedronate Therapy, North American Trial.

**Table 8:** Observed Rate of Nonvertebral Fracture by Trial (Per 1000 Person‐Years)

	Non‐DM			T2D		
Trial	Active	Placebo	IRR	Active	Placebo	IRR
FIT I	39.2	51.1	0.77	45.0	39.9	1.13
FIT II	27.0	29.4	0.92	19.3	41.2	0.47
BONE	34.3	31.2	1.10	32.9	38.8	0.85
IBAN IV	31.2	33.2	0.94	39.9	0	—
HIP	49.3	50.4	0.98	70.8	62.9	1.13
VERT‐MN	52.1	60.1	0.87	64.9	65.2	1.00
VERT‐NA	37.1	52.1	0.71	47.9	53.9	0.89
HORIZON PFT	28.8	37.0	0.78	21.2	50.6	0.42
HORIZON RFT	39.2	55.3	0.71	93.3	33.4	2.80
LOFT	15.8	21.7	0.73	21.4	22.7	0.94
WHI‐E	13.7	18.8	0.73	16.8	21.3	0.79
WHI‐EP	15.1	18.3	0.83	15.2	23.7	0.64
FREEDOM	22.9	30.2	0.76	37.9	22.5	1.69
PEARL	19.3	23.6	0.82	22.0	16.9	1.30
MORE	32.3	35.9	0.90	38.5	26.1	1.47

Rate = (# events/total person‐years)*1000 person‐years. BONE = Oral Ibandronate Osteoporosis Vertebral Fracture Trial in North America and Europe; FIT = Fracture Intervention Trial; FREEDOM = Fracture Reduction Evaluation of Denosumab in Osteoporosis Every 6 Months; HIP = Hip Intervention Program Study Group; HORIZON PFT = Health Outcomes and Reduced Incidence with Zoledronic Acid Once Yearly Pivotal Fracture Trial; HORIZON RFT = Health Outcomes and Reduced Incidence with Zoledronic Acid Once Yearly Recurrent Fracture Trial; IRR = incidence rate ratio; LOFT = Long‐term Odanacatib Fracture Trial; MORE = Multiple Outcomes of Raloxifene Evaluation; PEARL = Postmenopausal Evaluation and Risk‐Reduction with Lasofoxifene Study; VERT‐MN = Vertebral Efficacy with Risedronate Therapy, Multinational Trial; VERT‐NA = Vertebral Efficacy with Risedronate Therapy, North American Trial; WHI‐E = Women’s Health Initiative, Estrogen Arm; WHI‐EP = Women’s Health Initiative, Estrogen‐Progestin Arm.

**Table 9:** Observed Rate of All Fracture by Trial (Per 1000 Person‐Years)

	Non‐DM			T2D		
Trial	Active	Placebo	IRR	Active	Placebo	IRR
FIT I	66.2	103.1	0.64	81.9	73.8	1.11
FIT II	31.8	36.8	0.86	22.5	50.3	0.45
BONE	51.9	66.3	0.78	69.6	61.2	1.14
IBAN IV	64.9	72.7	0.89	60.2	0	—
HIP	75.5	86.3	0.87	80.4	95.2	0.84
VERT‐MN	119.5	180.1	0.66	64.9	205.8	0.32
VERT‐NA	79.2	104.4	0.76	83.8	116.7	0.72
HORIZON PFT	42.0	73.9	0.57	31.7	86.8	0.36
HORIZON RFT	46.3	73.5	0.63	93.3	33.4	2.80
LOFT	25.3	44.0	0.58	31.4	39.3	0.80
WHI‐E	15.0	20.8	0.72	17.1	24.1	0.71
WHI‐EP	16.7	20.4	0.82	16.6	25.0	0.67
FREEDOM	30.3	53.0	0.57	44.7	53.8	0.83
PEARL	31.8	43.6	0.73	34.0	30.6	1.11
MORE	49.3	66.2	0.75	41.9	54.6	0.77

Rate = (# events/total person‐years)*1000 person‐years. BONE = Oral Ibandronate Osteoporosis Vertebral Fracture Trial in North America and Europe; FIT = Fracture Intervention Trial; FREEDOM = Fracture Reduction Evaluation of Denosumab in Osteoporosis Every 6 Months; HIP = Hip Intervention Program Study Group; HORIZON PFT = Health Outcomes and Reduced Incidence with Zoledronic Acid Once Yearly Pivotal Fracture Trial; HORIZON RFT = Health Outcomes and Reduced Incidence with Zoledronic Acid Once Yearly Recurrent Fracture Trial; IRR = incidence rate ratio; LOFT = Long‐term Odanacatib Fracture Trial; MORE = Multiple Outcomes of Raloxifene Evaluation; PEARL = Postmenopausal Evaluation and Risk‐Reduction with Lasofoxifene Study; VERT‐MN = Vertebral Efficacy with Risedronate Therapy, Multinational Trial; VERT‐NA = Vertebral Efficacy with Risedronate Therapy, North American Trial; WHI‐E = Women’s Health Initiative, Estrogen Arm; WHI‐EP = Women’s Health Initiative, Estrogen‐Progestin Arm.

**Table 10:** Observed Rate of Hip Fracture by Trial (Per 1000 Person‐Years)

	Non‐DM			T2D		
Trial	Active	Placebo	IRR	Active	Placebo	IRR
FIT I	3.9	7.6	0.51	0	9.7	—
FIT II	1.9	2.3	0.81	3.1	6.9	0.45
BONE	3.7	1.7	2.12	0	0	—
IBAN IV	2.3	4.9	0.47	19.3	0	—
HIP	8.9	13.1	0.68	26.9	8.7	3.08
VERT‐MN	8.6	10.0	0.86	0	0	—
VERT‐NA	5.0	3.4	1.46	0	0	—
HORIZON PFT	4.8	7.9	0.61	5.5	13.4	0.41
HORIZON RFT	11.0	16.5	0.67	29.4	0	—
LOFT	2.3	4.4	0.52	4.4	8.9	0.50
WHI‐E	1.2	1.9	0.65	2.3	2.1	1.07
WHI‐EP	1.6	2.0	0.79	1.8	3.0	0.59
FREEDOM	2.5	4.1	0.62	1.2	3.9	0.32
PEARL	2.0	2.9	0.70	4.0	0	—
MORE	3.2	0.9	3.52	6.1	0	—

Rate = (# events/total person‐years)*1000 person‐years. BONE = Oral Ibandronate Osteoporosis Vertebral Fracture Trial in North America and Europe; FIT = Fracture Intervention Trial; FREEDOM = Fracture Reduction Evaluation of Denosumab in Osteoporosis Every 6 Months; HIP = Hip Intervention Program Study Group; HORIZON PFT = Health Outcomes and Reduced Incidence with Zoledronic Acid Once Yearly Pivotal Fracture Trial; HORIZON RFT = Health Outcomes and Reduced Incidence with Zoledronic Acid Once Yearly Recurrent Fracture Trial; IRR = incidence rate ratio; LOFT = Long‐term Odanacatib Fracture Trial; MORE = Multiple Outcomes of Raloxifene Evaluation; PEARL = Postmenopausal Evaluation and Risk‐Reduction with Lasofoxifene Study; VERT‐MN = Vertebral Efficacy with Risedronate Therapy, Multinational Trial; VERT‐NA = Vertebral Efficacy with Risedronate Therapy, North American Trial; WHI‐E = Women’s Health Initiative, Estrogen Arm; WHI‐EP = Women’s Health Initiative, Estrogen‐Progestin Arm.

## Discussion

The key finding of this study was that the anti‐fracture efficacy of antiresorptive therapies, considered as a group, was similar in people without and with T2D for all four types of fractures studied (vertebral, hip, nonvertebral, and “all” fractures). In addition, anti‐resorptive therapies resulted in similar improvements in total hip and femoral neck BMD and similar reductions in both formation (P1NP) and resorption markers (CTX and NTX) for T2D and non‐DM patients.

The observation that those with T2D had higher BMI at baseline was expected. The differences in age and gender were very small and unlikely to have affected the results. It was odd that there was a lower prevalence of vertebral fracture at baseline in those with T2D, although this finding is in keeping with a recent meta‐analysis that reported no difference in vertebral fracture prevalence between those with and without T2D.^(^ ^29^ ^)^ The generally higher baseline BMD in T2D was consistent with prior work.^(^ ^2^ ^)^ The lower BTMs at baseline was also consistent with prior reports.^(^ ^3^ ^)^ Nonetheless, it should be remembered that the participants enrolled in these trials, both with and without diabetes, are not representative of the general population because entry criteria for these trials generally included only those at higher fracture risk so we might find differences we were not expecting based on comparisons in broader populations.

There is some evidence that higher baseline BTM levels are associated with greater treatment‐related increase in BMD with antiresorptive treatments such as alendronate and menopausal hormone therapy.^(^ ^30^, ^31^ ^)^ However, the BTM levels in T2D this study were only reduced by about 10% (Table 2) in contrast to those in T2D as a whole in whom BTMs are decreased by about 25%.^(^ ^3^ ^)^

In the SABRE study, we previously reported that baseline BTM^(^ ^32^ ^)^ and the change in BTM^(^ ^8^ ^)^ are both strong predictors of fracture risk reduction. Thus, it was no surprise that there was no difference overall or in the bisphosphonate group alone that fracture risk reduction for all fracture types did not differ between those without or with T2D.

The results from a few individual trials were inconsistent with the overall findings. In particular, we found a negative effect of denosumab on nonvertebral fractures among T2D, a finding that is consistent with a prior analysis.^(^ ^7^ ^)^ However, denosumab was equally effective in reducing vertebral fracture risk in those with and without T2D. We also observed a lesser effect of odanacatib on all fractures in those with T2D. For odanacatib, the fracture results are consistent with findings for BMD, namely that total hip BMD and femoral neck BMD gains were smaller in those with T2D compared with non‐DM. We do have to be cautious about overinterpretation of our findings as we calculated 42 *p* values for interaction in the three figures showing fracture risk reduction within trials and we might expect some false positives.

We considered whether there might be any reason for denosumab appearing to have a negative effect on nonvertebral fractures in T2D. A possible explanation is that denosumab reduces BTM to unfavorable levels in T2D. BTMs are lower in T2D and the poorer the diabetes control, the lower the BTM.^(^ ^3^ ^)^ Denosumab is the most potent antiresorptive treatment with decreases in CTX of more than 90% on average.^(^ ^33^ ^)^ We lacked BTM data for denosumab so we were not able to evaluate this hypothesis.

What does this mean with respect to treatment of osteoporosis for patients with T2D in clinical practice? The usual lifestyle measures for the prevention of fractures, such as exercise and a diet adequate in calcium and vitamin D are also important in T2D. In addition, prevention of fractures is helped by good diabetes control and the avoidance of diabetes drugs that might increase fracture risk, such as glitazones.^(^ ^34^ ^)^ Our results indicate that anti‐resorptive drugs tested for osteoporosis appear to be equally effective in those with and without T2D. Concerns regarding possible reduced anti‐fracture efficacy in T2D due to lower BTM in T2D were not borne out in our results. The bisphosphonates are the first line treatment for osteoporosis in broader populations, and our results indicate that these drugs can also be used effectively in patients with T2D. Considering other specific classes of anti‐resorptive therapy, our results indicate denosumab would be appropriate in a patient at high risk of vertebral fracture but may not be so appropriate in a patient with T2D at high risk of nonvertebral fractures. It would be very helpful to have a clinical trial of denosumab in T2D to determine whether increased nonvertebral fracture risk was a spurious finding.

We did not study anabolic therapies due to the low number of trials in the SABRE database. To date, only observational studies are available on the anti‐fracture efficacy in T2D compared with non‐DM patients. An analysis of four observational studies of 8828 patients indicates that teriparatide may reduce the risk of fracture in those with T2D.^(^ ^35^ ^)^ A post hoc analysis of the Abaloparatide Comparator Trial In Vertebral Endpoints (ACTIVE) study indicates that abaloparatide increases BMD in postmenopausal women with T2D to a similar extent as in those without T2D.^(^ ^36^ ^)^

The major strength of our analysis was that the trials we included were all randomized controlled clinical trials and so this avoids confounding by indication. Although the definition used for diabetes likely varied across trials, it was consistent for the active and placebo groups within a trial. The definition of diabetes often (but not always) relied on self‐report. However, in the Women's Health Initiative study, the positive predictive value of self‐report of having T2D was 92% and the negative predictive value of not having diabetes was 95%,^(^ ^37^ ^)^ so misdiagnosis was unlikely to have been an important issue.

Our study had weaknesses. We examined many associations, thus increasing the risk of false‐positive results; for interaction terms in the figures alone we reported at least 80 *p* values. Nominally statistically significant *p* values should be interpreted with caution in the context of multiple comparisons. Also, the analysis was not planned, it was post hoc. We did not have fasting plasma glucose in all studies and so some patients could have been misclassified. We were not able to distinguish between type 1 diabetes (T1D) and T2D. However, given the much higher prevalence of T2D among older adults, we have interpreted our results as applying to T2D. Our results may not generalize to those with T1D.

In conclusion, the bisphosphonates and most other antiresorptive drugs appear to have similar efficacy in individuals with and without diabetes, whether this is evaluated by fracture risk reduction, increases in BMD, or reductions in BTM.

## AUTHOR CONTRIBUTIONS

**Richard Eastell:** Conceptualization; investigation; methodology; writing – original draft; writing – review and editing. **Eric Vittinghoff:** Formal analysis; writing – review and editing. **Li‐Yung Lui:** Formal analysis; writing – review and editing. **Susan K. Ewing:** Data curation; formal analysis; writing – review and editing. **Ann V. Schwartz:** Conceptualization; supervision; writing – review and editing. **Douglas C Bauer:** Investigation; writing – review and editing. **Dennis M. Black:** Conceptualization; data curation; funding acquisition; writing – review and editing. **Mary L. Bouxsein:** Conceptualization; funding acquisition; visualization; writing – review and editing.

## Conflicts of Interest

RE: reports grants from Amgen, grants and personal fees from Immunodiagnostic Systems, grants from Alexion, grants and personal fees from Roche, personal fees from Eli Lilly, personal fees from GSK Nutrition, personal fees from Mereo, personal fees from Sandoz, grants and personal fees from Nittobo, personal fees from AbbVie, personal fees from Samsung, personal fees from Haoma Medica, personal fees from Elsevier, personal fees from CL Bio, personal fees from FNIH, personal fees from Viking, outside the submitted work. EV: reports salary support from FNIH, during the conduct of the study. DMB: personal fees from Merck, personal fees from Amgen, personal fees from Asahi‐Kasei, personal fees from Effx, during the conduct of the study; personal fees from Eli Lilly, personal fees from University of Pittsburgh, outside the submitted work. MLB: received a grant from the Foundation for the NIH in relation to the submitted work, and grants from Amgen and Radius Pharma unrelated to this work; personal fees from Beryl Health, Agnovos and Keros Therapeutics. LYL, SKE, DCB, and AVS have nothing to disclose.

### PEER REVIEW

The peer review history for this article is available at https://publons.com/publon/10.1002/jbmr.4697.

## Data Availability

As stated above, all study data were acquired by requesting IPD from study sponsors. An overarching data use agreement was created between all parties and individual data use agreements were created between individual study sponsors, FNIH and UCSF. Per the data sharing agreements that we have with each sponsor, the data can be used for surrogate marker analyses including any surrogate qualification processes with regulatory authorities. However, other uses of the data are restricted by this agreement and UCSF is not allowed to share the data.
